# Melodic Intonation Therapy: Back to Basics for Future Research

**DOI:** 10.3389/fneur.2014.00007

**Published:** 2014-01-28

**Authors:** Anna Zumbansen, Isabelle Peretz, Sylvie Hébert

**Affiliations:** ^1^Faculty of Medicine, School of Speech Pathology and Audiology, Université de Montréal, Montréal, QC, Canada; ^2^BRAMS, International Laboratory for Brain, Music and Sound Research, Université de Montréal, Montréal, QC, Canada; ^3^Department of Psychology, Université de Montréal, Montréal, QC, Canada

**Keywords:** melodic intonation therapy, aphasia, speech disorders, apraxia of speech, treatment, rehabilitation, speech therapy, music therapy

## Abstract

We present a critical review of the literature on melodic intonation therapy (MIT), one of the most formalized treatments used by speech-language therapist in Broca’s aphasia. We suggest basic clarifications to enhance the scientific support of this promising treatment. First, therapeutic protocols using singing as a speech facilitation technique are not necessarily MIT. The goal of MIT is to restore propositional speech. The rationale is that patients can learn a new way to speak through singing by using language-capable regions of the right cerebral hemisphere. Eventually, patients are supposed to use this way of speaking permanently but not to sing overtly. We argue that many treatment programs covered in systematic reviews on MIT’s efficacy do not match MIT’s therapeutic goal and rationale. Critically, we identified two main variations of MIT: the French *thérapie mélodique et rythmée* (TMR) that trains patients to use singing overtly as a facilitation technique in case of speech struggle and *palliative versions of MIT* that help patients with the most severe expressive deficits produce a limited set of useful, readymade phrases. Second, we distinguish between the immediate effect of singing on speech production and the long-term effect of the entire program on language recovery. Many results in the MIT literature can be explained by this temporal perspective. Finally, we propose that MIT can be viewed as a treatment of apraxia of speech more than aphasia. This issue should be explored in future experimental studies.

Melodic intonation therapy [MIT; (1, 2)] is a treatment program used by speech-language pathologists for the rehabilitation of patients with speech production disorders. At the first levels of the MIT program, musical components are used to facilitate verbal expression. Typically, the clinician asks the patient to produce everyday sentences in a singing-like manner that exaggerates the natural prosody (pitch variation and rhythmic features) while tapping with the left hand on each syllable. MIT was initially based on the hypothesis that music processing regions of the right cerebral hemisphere had language capabilities, and that they could potentially compensate for damaged left hemisphere language regions. The participation of the left hand was thought to help the intoned-speech facilitation technique stimulate language-capable areas in the right hemisphere (3). The program is described in detail in a manual and a demonstration video (4, 5). The American Academy of Neurology has rated the MIT as promising for brain-damaged patients who meet the criteria for Broca’s aphasia (6).

Because MIT is one of the most formalized treatments in speech-language therapy (6), it is particularly well suited for scientific study. However, 40 years after its original publication, systematic reviews still comment on the low quality of the MIT efficacy studies and raise questions regarding the treatment mechanisms (7, 8). In fact, in addition to intoned speech, MIT includes various other therapeutic techniques (e.g., rhythmic speech, auditory and visual cueing, production of formulaic expressions) that make the study of MIT’s mechanisms challenging.

There is no consensus in the literature on the definition of MIT, which has sometimes been reduced to the intoned-speech facilitation technique rather than the entire program, similar to describing a recipe as one of the ingredients. Consequently, so-called MIT programs vary widely. Moreover, it is important to distinguish between the immediate effects of the technique on speech accuracy and the longer-term effects of the entire program on language recovery. In our review, we discuss the implications of the main deviations from the original MIT. A second objective is to consider the plausible mechanisms involved in the MIT and to suggest novel approaches in order to understand better these mechanisms.

## MIT and Its Variations

First, we will define the basic goal of MIT according to its designers and how they developed MIT’s rationale with this in mind (1–3).

### Goal of the original MIT

Melodic intonation therapy was developed to improve propositional language (1, 2), or the generative and controlled language production that people use in everyday life to express their ideas (9). Propositional language requires an assemblage of structures according to a set of phonological, morphological, and grammatical rules and in accord with a lexicon. It is opposed to non-propositional language (also referred to as automatic, or formulaic language), which is also used in everyday life, but which consists of a repertory of readymade and over-learned expressions [for example, idioms, proverbs, and even longer material in prayers or in songs; see Ref. (10) for a review]. This type of non-generative verbalization is known to be relatively preserved in Broca’s aphasia (10). MIT uses everyday sentences, thus, a part of the verbal material falls into the formulaic category. Speech formulas (e.g., “good morning,” “how are you?”) and sentence stems (e.g., “I am,” “I want”) are often used, especially in the first levels of the program (4, 5). Formulaic expressions are relevant in speech and language therapy because they represent a significant proportion of spoken phrases in daily living [at least 25% in American English; (11)]. Moreover, this verbal material may be of high value to keep the patients motivated throughout the therapy because it is easier to produce than propositional phrases. In sum, MIT uses both formulaic and non-formulaic verbal material although the goal remains to improve propositional speech. It remains unclear, however, how training on formulaic verbal material results in improvement in propositional speech.

Because MIT targets generative speech, the American Academy of Neurology recommends that efficacy studies should measure the effect of the program on non-trained material, standard language tests, and spontaneous connected speech (6). Ideally, the language assessment should be sensitive to capture the proportion of conventionalized speech formulas in spontaneous utterances.

### Rationale of the original MIT program

The original MIT is based on the observation that people with aphasia are able to sing familiar songs (12), but that a therapy based on this kind of activity has no impact on the recovery of propositional language (2). Nevertheless, it was thought that regions of the right hemisphere involved in music processing could take over the homolog-damaged regions of the left hemisphere if they were properly stimulated (2). In behavioral terms, the idea is that patients can learn a permanent new way of speaking through singing.

**Figure 1:**
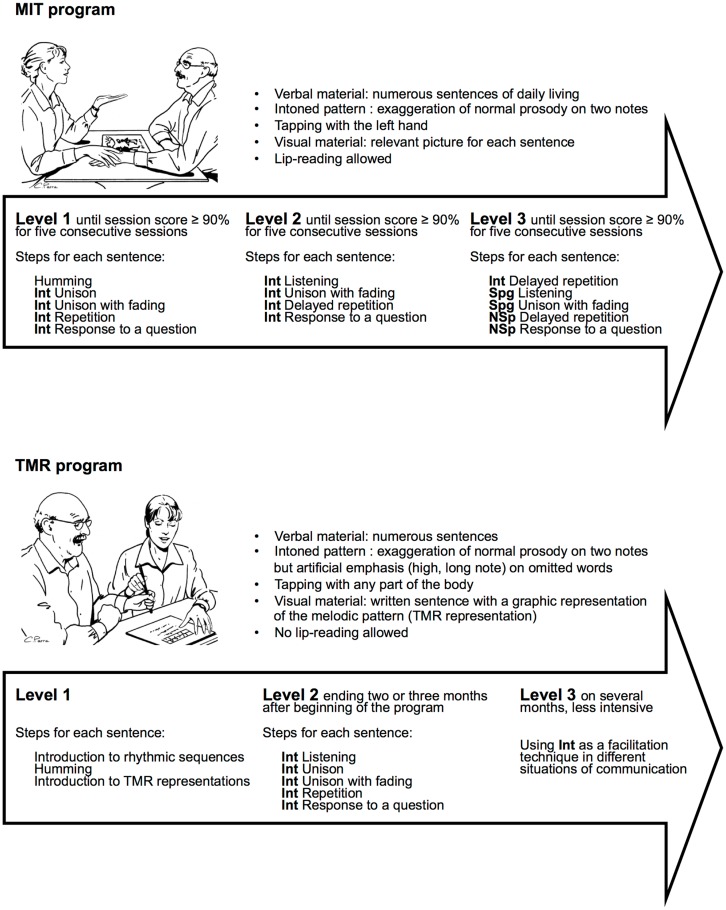
**Comparison between original MIT (5) and TMR (Thérapie mélodique et rythmée, 28), a French version of MIT**. Int, intoned item; Spg, Sprechgesang item (halfway between normal speech and intoned speech, i.e., rhythmically emphasized prosody); NSp, item in normal speech.

The MIT guides the patient to gradually adopt this new way of speaking (Figure 1). In the first levels, the patient learns to speak everyday sentences using a facilitation technique called *intoned speech*. Intoned speech is made of pitch and rhythm features based on the exaggeration of normal speech prosody (13, 14). The varying pitch of speech is reduced to two constant pitches, which are two musical notes usually separated by a third or a fourth. The high pitch is used for stressed syllables and the low pitch for unstressed syllables. The rhythm of normal speech is also musically stylized: the tempo is lengthened, the rhythmic pattern is reduced to quarter and eighth notes and the loudness is increased on stressed syllables.

Because the patient learns a new way to speak, and not a set of sentences, the verbal material must be copious, varied, and presented so as to avoid the use of rote memory (13). The treatment intensity is adapted to this type of learning: frequent sessions (at least 3 week) for 3–6 weeks (6).

The verbal material is worked out step by step, using left hand tapping in addition to intoned speech to stimulate the right hemisphere (3). The procedure begins with the easiest condition (i.e., intoning in unison with the clinician) and progresses to more autonomous production (i.e., responses to questions). When the patient has mastered overt intoned speech, the last program level is introduced, where patients progressively learn to drop the musical components from their speech output. To do so, the clinician uses another facilitation technique called *Sprechgesang*. Sprechgesang has the same rhythmic features than intoned speech but the constant pitches are replaced by a more varying pitch, more similar to speech than singing. Thus, Sprechgesang is half-way between singing and speech (13).

### Variations of MIT and implications in efficacy studies

#### The intoned-speech facilitation technique vs. the MIT program

Intoned speech is the main facilitation technique of MIT. It has been extensively described by Sparks and colleagues (13, 14) to help clinician implementing MIT in their clinical practice. Because MIT has sometimes been reduced to the intoned-speech facilitation technique rather than considered as an entire program, the distinction between a facilitation technique and a treatment program is not always made. Speech and language therapists use different techniques to facilitate verbal output, such as phonemic cuing, semantic cuing, simultaneous imitation of speech and articulatory movements (i.e., lip-reading). The effect of a facilitation technique is immediate and highly dependent on the facilitating condition. In speech and language therapy, a treatment program is a plan of actions designed to attain a specific therapeutic goal. In contrast to a facilitation technique, the effect of a treatment program reveals itself with time, as measured outcomes associated with the therapeutic goals. When designing a therapy, clinicians choose which facilitation techniques are relevant for the patient, when to use them, and to which verbal material they are applied.

Melodic intonation therapy is a treatment program that combines several facilitation techniques, including intoned speech, Sprechgesang (i.e., rhythmically emphasized prosody), unison production with the clinician, and lip-reading (Figure 1). Although intoned speech is the main facilitation technique, it could be included in other treatment programs having different goals, rationale, and design.

In the following section, we review some variations made to the original MIT program and its facilitation technique.

#### Analysis of MIT’s modifications in two recent systematic reviews

We examined 14 publications (Table 1) identified as MIT efficacy studies in two recent systematic reviews (7, 8). We found that all the treatments shared two characteristics. First, they guided patients to produce some verbal material on a melody. Second, there is always a progression from the most facilitating and assisted condition to a more autonomous production of intoned speech, which usually include unison, unison with fading, immediate repetition, delayed repetition, and response to a question. However, of the 14 publications, we found only 5 in which the programs matched MIT therapeutic goals and rationale (2, 15–18). We detail below four types of modifications that can change the very basics of the original MIT.

**Table 1:** **Some characteristics of the therapeutic protocols regarded as MIT in systematic reviews**.

Authors and MIT version *(original, TMR, palliative, or other)*	*N*	Verbal material	Principal outcome	Schedule	Intoned items	Tapping
Bonakdarpour et al. (15), *original*	7	Numerous sentences	Scores on standard language test; connected speech analysis	3–4 days/week, over 4 weeks; original MIT	Exaggeration of normal prosody	Left hand tapping
Naeser and Helm-Estabrooks (16), *original*	8	Numerous sentences	Scores on standard language test	Over 1–8 weeks; original MIT	Exaggeration of normal prosody	Left hand tapping
Schlaug et al. (17), *original*	2	Numerous sentences	Scores on standard language test; connected speech analysis	90 min, 5 days/week, over 8 weeks; original MIT	Exaggeration of normal prosody	Left hand tapping
Schlaug et al. (18), *original*	6	Numerous sentences	Scores on standard language test; connected speech analysis	75 Sessions; original MIT	Exaggeration of normal prosody	Left hand tapping
Sparks et al. (2), *original*	8	Numerous sentences	Scores on standard language test; connected speech analysis	Over 3 months; original MIT	Exaggeration of normal prosody	Left hand tapping
Belin et al. (19), *TMR*	7	Numerous sentences	Scores on standard language test	Over 37–42 months; no return to normal speech	Exaggeration of normal prosody but artificial emphasis (high note) on omitted words	Tapping (any part of the body)
Baker (20), *palliative*	2	Limited set of sentences	Number of sentences recalled	30 min, 3–8 days/week, over 4–27 months; no return to normal speech	Specific musical line and accompaniment for each trained sentence (mnemonic cue)	No tapping
Goldfarb and Bader (21), *palliative*	1	Limited set of sentences	Intelligibility of trained sentences	60 min, 7 days/week; return to normal speech	Exaggeration of normal prosody	Left hand tapping
Hough (22), *palliative*	1	Limited set of sentences	Intelligibility of trained sentences	3 days/week, over 8 weeks; no return to normal speech	Exaggeration of normal prosody	No tapping
Springer et al. (23), *palliative*	12	Limited set of sentences with Wh-questions and prepositions	Use of Wh-questions and prepositions in non-trained sentences	60 min, 3–4 days/week, over 2 weeks; MIT’s facilitation technique used in a different therapeutic program	Exaggeration of normal prosody	Hand tapping (no more precision)
Wilson et al. (24), *palliative*	1	Limited set of sentences	Intelligibility of trained sentences	2 days/week, over 4 weeks; no return to normal speech	Exaggeration of normal prosody	Left hand tapping
Buttet and Aubert (25), *other*	7	Numerous sentences	Clinical judgment of propositional language	Up to 20 min, 1–2 sessions/day, 4–5 days/week, over 2–8 months; MIT’s facilitation technique used to start sessions of different therapeutic programs	Exaggeration of normal prosody	Left hand tapping
Marshall and Holtzapple (26), *other*	2	Numerous sentences made of redundant parts and various core words	Scores on standard language test	60 min, 3 days/week, over 3 months; no return to normal speech	Exaggeration of normal prosody	Hand tapping (no more precision)
Popovici et al. (27), *other*	80 (+80 controls)	Numerous sentences	Scores on standard language test	60–120 min, 7 days/week, over 2–4 weeks; no return to normal speech	Exaggeration of normal prosody	Hand tapping (no more precision)

##### Verbal material and outcome measures

Four studies (20–24) measured improvements on trained material following a program, where patients were given intensive training in order to produce a limited set of sentences. The results do not allow drawing generalized interpretations concerning generative language. Unlike the original MIT, the goal of these rehabilitation protocols is not to restore generative language. Instead, the goal is to provide severely impaired patients with a few readymade sentences that can be quickly used for basic communication in everyday situations. In this perspective, the protocols may be viewed as *palliative* versions of MIT, and certain generalizations may be made. For example, Hough (22) observed improvements on non-trained items and standard language tests, although they were not statistically analyzed. Thus, palliative versions of MIT may be an interesting option for patients who do not reach the higher levels of the original program. These protocols could also be tested with the considerable subgroup of aphasic patients who do not fit the strict candidacy criteria for original MIT (2, 6).

##### Therapeutic program schedule

In MIT, the final treatment sessions are designed to help the patient gradually return to normal speech. The end goal is permanent use of a new way of speaking through melodic intonation, but not intoned speech output. Five publications (20, 24, 26, 27) do not mention whether or how a return to normal speech was planned. In a French version of the MIT, *thérapie mélodique et rythmée* [TMR; (28)], used by Belin and colleagues (19), there is no guided return to normal speech. The patient learns to overtly resort to the intoned technique as occasional assistance in case of trouble. This approach is consistent with the recommended TMR schedule (Figure 1), which ends with an extended, less intensive period where the patient is trained to use the facilitation technique from time to time in different daily situations.

In two other studies (23, 25), only the intoned technique was used for its immediate facilitation effect in treatment programs that otherwise differed completely from the original MIT.

##### Construction of intoned items

In MIT, the melody used to intone sentences consists of an exaggeration of the normal prosody. The musical quality is drawn directly from typical prosody, because it is thought to activate musical regions in the right cerebral hemisphere that could have language capability. However, in the sung items of TMR [Ref. (28), used in Ref. (19)], some typically non-accented words are intentionally emphasized in the melody because they are often omitted in aphasic speech (e.g., function words, such as articles and prepositions). According to Van Eeckhout and Bhatt (28), because the construction of intoned items is based on linguistic and not musical criteria, this departure from the original MIT is in line with TMR’s rationale, which does not assume right hemisphere involvement in language recovery. The principle of TMR is instead behavioral: the prosodic components are thought to play an important role in verbal communication, and focusing on the melodic aspect of speech can decondition patients’ preoccupations with their speech production deficit (28).

In one of the palliative versions of MIT (20), each trained phrase is sung using a more complex melody than the typical two-pitch melodic intonation of the original MIT, and the clinician uses an instrument to create harmony. The musical element is a mnemonic cue that helps patients recall trained sentences rather than facilitating general verbal production, as in the original MIT.

##### Tapping

Tapping with the left hand is related to the presumed right hemisphere involvement in the original MIT. However, tapping was not included in the treatment in two studies (20, 22), and three other publications (23, 26, 27) did not mention, which hand was used to tap the rhythmic component of the melody. TMR also allows using parts of the body other than the left hand, as this modification of the protocol does not consider cerebral hemisphere dominance for the treatment effect (28).

### Concluding remarks

A number of MIT modifications may reflect adaptations to the various personal and clinical profiles in people with aphasia, even when the deficit is categorized as Broca’s aphasia (20, 26). In fact, the authors of the original MIT encouraged clinicians to adapt the protocol to specific patient needs (14). However, the above listed modifications have basic therapeutic implications. Two main variation of MIT can be identified according to their therapeutic goals: palliative MIT and TMR. Instead of learning a new internalized way to speak, palliative versions of MIT train patients to produce a limited set of sentences, and TMR train patients to use a facilitation technique overtly when needed. Thus, efficacy reports on modified MIT versions cannot be considered evidence of the efficacy of the MIT. To date, the original MIT has demonstrated the best results in treating Broca’s aphasia, but this is supported by only five studies in a total of 31 patients (2, 15–18). Two randomized controlled clinical trials are currently underway in the United States (29) and Netherlands (30), and will hopefully provide more solid support for MIT’s efficacy.

Some modifications (e.g., construction of intoned items based on linguistic rather than melodic features, no use of left hand tapping) are also associated with a departure from the rationale of the original MIT. At least theoretically, these modifications could have an impact on the mechanisms by which MIT works on language recovery.

## Mechanisms

### Role of the right cerebral hemisphere

The initial hypothesis concerning the role of the right cerebral hemisphere to the MIT efficacy remains debated. Table 2 summarizes the brain imaging studies that have tested this hypothesis in a total of 22 participants with aphasia. Whereas some results are supportive (17, 18, 31), others fail to support MIT’s rationale (32), and some researchers have concluded that MIT instead promotes left peri-lesional activation (19, 33, 34). Besides the fact that these studies used different functional imaging techniques, at least four major factors could explain these inconsistent findings.

**Table 2:** **Imaging studies on brain substrates in MIT**.

Authors	Imaging technique	MIT version (*original, TMR, palliative or other*)	*N*	Participants’ aphasia type	Time of acquisition	Imaging paradigm (contrast if applicable)	Involvement of LH and RH
Schlaug et al. (17)	fMRI	*Original*	2	Chronic Broca’s aphasia	Pre and post	Repetition of sentences either with normal prosody or intoned (normal speech vs. silence)	Pre-: RH and LH
							Pre- and post-: More RH than pre
Schlaug et al. (18)	DTI	*Original*	6	Chronic Broca’s aphasia	Pre and post	n/a	Pre- and post-: Plasticity in the RH arcuate fasciculus
Laine et al. (32)	SPECT	*Original*	3	1 Chronic Broca’s aphasia; 1 chronic mixed non-fluent aphasia; 1 chronic Wernicke’s aphasia	Pre	Repetition of words and sentences either with normal prosody or intoned (intoned vs. normal speech)	Pre-: More LH than RH in subject with Broca’s aphasia; mixed lateralization in subject with mixed non-fluent aphasia; no difference in patient with Wernicke’s aphasia
Belin et al. (19)	PET	*TMR*	7	2 Chronic Broca’s aphasia; 5 chronic global aphasia	Post	Repetition of sentences either with normal prosody or intoned (normal speech vs. silence; intoned vs. normal speech)	Post-: More RH than LH (normal speech vs. silence);
							more LH than RH (intoned vs. normal speech)
Sandt-Koenderman et al. (34)	fMRI	*Palliative*	1	Broca’s aphasia in the subacute stage post stroke	Pre and post	Lexical decision task with non-language input or verbal inputs either with normal prosody or intoned (normal speech vs. non-language; intoned vs. normal speech)	Pre- and post-: More LH than pre (normal speech vs. non-language); no difference (intoned vs. normal speech)
Breier et al. (33)	MEG	*Palliative*	2	Chronic mixed aphasia	Pre and post	Covert action naming task	Pre-: More LH than RH
							Pre- and post-: More LH than pre
Zipse et al. (31)	fMRI and DTI	*Other (original MIT added with two additional techniques)*	1	Chronic Broca’s aphasia	Pre and post	fMRI: Repetition of sentences either with normal prosody or intoned (normal speech vs. silence); DTI: n/a	Pre-: RH and LH Pre- and post-: More RH than pre (fMRI); plasticity in the RH arcuate fasciculus (DTI)

First, only three of the seven studies used the original MIT (17, 18, 31). As discussed above, some variations in protocol changed the original treatment goal and rationale. Some of these modifications may involve different brain substrates than the original MIT. In fact, no study supporting the left hemisphere hypothesis has used the original MIT protocol.

Second, not all studies used pre- and post-therapy acquisition. Belin and colleagues (19) only reported post-treatment data in a selected group of participants who showed clear improvement on language tests after TMR. Laine and colleagues (32) acquired pre-treatment data only. In both studies, pre- and post-treatment differences in brain activation patterns during speech production are not reported. Thus, the results cannot be associated with the treatment effect.

Third, the functional neuroimaging paradigms differed across studies. One study used a covert action naming task (33), another used a lexical decision task (34), and the remaining studies used repetition of target words or phrases (17, 19, 31, 32). Even within a single aphasic patient, lateralization in brain activation following therapy varies depending on the language task (35). Therefore, caution must be taken when comparing studies using different brain imaging paradigms.

Individual differences constitute another important factor to consider. Lateralization of brain activation related to language tasks likely varies depending on the time elapsed since a stroke (36). Moreover, it is unclear if right hemisphere activation is correlated with language improvements (37). For example, Zipse and colleagues (31) recently reported in a successfully treated teenager with Broca’s aphasia that brain activations in the right hemisphere during a spoken repetition task increased with the first 40 MIT sessions and decreased 40 sessions later. This was interpreted as the result of automatization of the MIT way of speaking. Brain reorganization after stroke also highly depends on the site and extent of lesions (38). According to the hierarchical model of brain compensation strategies after stroke (39), right hemisphere areas can support some language recovery only if essential language areas of the left hemisphere are destroyed. Thus, much of the potential specific brain correlates of MIT can be masked by individual factors.

Despite the difficulty of properly comparing the different studies in terms of the brain substrates involved in MIT, three consistent observations may be made, based on four reports that used a similar repetition paradigm of bisyllabic words and/or short phrases with aphasic participants in the chronic stage (17, 19, 31, 32). Two publications found consistent right lateralized activation when contrasting normal speech with silence (17, 19). As healthy subjects usually show more activation in the left hemisphere under these conditions, Belin and colleagues interpreted this activation pattern as maladaptive, whereas Schlaug and colleagues considered it a beneficial cortical reorganization. However, a left lateralized activation pattern was reported when contrasting intoned with normal speech in participants with Broca’s aphasia (19, 32). This contrast is attributable to the effect of MIT’s main facilitation technique (i.e., intoned speech). Surprisingly, this finding does not support the idea that the use of musical components elicits more activation in the right hemisphere, although participants produced more words correctly when singing compared to natural speech, in French (19). Conversely, increased activation was found in the right hemisphere when comparing normal speech pre- and post-treatment (17, 31). This contrast is attributable to the overall effect of MIT as a program. In this case, results are in line with the original MIT rationale.

Some evidence point to an association of formulaic expressions with right frontotemporal areas, the right basal ganglia and, possibly, the right cerebellum (40–44). Although firm conclusions regarding these brain correlates are not possible to date, it has been proposed that the repeated training of formulaic verbal material could lead to the specific reinforcement of a right hemisphere circuits (45). At least theoretically, neuroimaging results previously attributed to singing could actually arise from the extensive use of conventionalized speech formulas. The formulaic status of the stimuli should be taken into account in the context of neuroimaging studies. Unfortunately, the stimuli used in brain imaging studies of MIT are usually neither provided nor described with regard to formulaicity. This certainly deserves more attention in future studies.

Thus, the role of the cerebral hemispheres in the MIT mechanism could be better determined by distinguishing the facilitation technique effect and the program effect. How these two effects interact remains to be understood, and could be explored within the broader issue of brain reorganization after stroke. To date, brain imaging data acquired pre- and post-MIT are only available for 12 participants (Table 2). Increased right hemisphere activation or white matter plasticity were found following original MIT in nine participants with chronic aphasia and large left hemisphere lesions (17, 18, 31) but Schlaug and colleagues (18) argue that using the right hemisphere for language processing might be the only option for such patients. Additional functional neuroimaging data using the same methodology are needed to fuel the debate on the cerebral correlates of the MIT effect. However, this debate should not overshadow research on other, more behavioral hypotheses of MIT mechanisms.

### Mechanisms of the intoned-speech facilitation technique (cross-sectional studies)

#### Singing along rather than singing alone

One of the most compelling reasons for using singing in a speech facilitation technique in MIT (1) is that patients with severe non-fluent aphasia were clinically described to produce words in familiar songs although they had otherwise extremely reduced speech output (46, 47). Further reports (12, 48, 49) gave rise to the general idea that singing would provide an effective way for people with aphasia to pronounce words. More recently, however, controlled quantitative studies have questioned this idea, having failed to demonstrate the superiority of the singing condition over normal speech for word production in non-fluent aphasia [(50) (12 subjects); (51) (1 subject); (52) (1 subject); (53) (17 subjects)]. It was hypothesized that the automatic status of lyrics in over-learned songs could account for the earlier clinical descriptions of better verbal production in familiar songs compared to spontaneous speech.

In a thorough study of the facilitation effect of singing in aphasia, Racette and colleagues (54) measured the number of correctly produced words by eight participants with non-fluent aphasia in a repetition task involving target sentences composed of familiar or non-familiar verbal material. They also compared verbal production when singing or speaking, and in unison with an auditory model or alone. Results showed no significant difference between singing and speaking alone, whether the verbal material was familiar or not. The only condition that appeared to effectively facilitate word production in patients with non-fluent aphasia in this study was *singing along* (also referred to as *choral singing*, *unison singing*, or *song shadowing*). Interestingly, this is the condition that the authors of MIT deemed the most clinically facilitating for teaching the intoned-speech technique to patients.

Racette and colleagues further discuss two results of their study: (1) the finding of *singing along over singing alone* and (2) the finding of *singing along over speaking along*. According to the authors, singing in unison promoted better speech output than singing alone, because it allowed patients to synchronize with a stable model, reducing the task-associated memory load compared to sung repetition alone. They speculated that unison production had a positive impact on the accuracy of motor speech planning and performance through the involvement of the mirror neuron system (55, 56) or the auditory-motor interface (57–59). These two theoretical systems suppose a direct relationship between perception and action in language (56, 58) and music (57). With regard to the second contrast (i.e., singing along over speaking along), Racette and colleagues (54) suggested that the ability to imitate in synchrony with an auditory model could be easier when the words are sung rather than spoken, because sung lyrics are more regular in rhythm than spoken words, and greater temporal regularity allows better synchronization (60). However, syllable duration was not controlled in this experiment since the stimuli were made of natural singing or natural speech. The sung syllables were almost twice as long as the spoken syllables. Consequently, the superiority of choral singing over choral speech may actually depend on differences in syllable duration. The study of Stahl and colleagues (53) provides support for this view: controlling for syllable duration, they did not find an effect of choral singing over choral speech in a group of 17 patients with non-fluent aphasia.

In the next section, we address the importance of rhythmic aspects (i.e., rhythmicity, tempo, and syllable duration) in MIT’s main facilitation technique.

#### Contribution of rhythm and pitch variation in the intoned-speech facilitation technique

Several studies have attempted to determine the relative contribution of the pitch and rhythmic components as used in MIT’s main facilitation technique [(61) (2 subjects); (62) (5 subjects); (53) (17 subjects)] and found that production of target sentences during MIT sessions appears facilitated by rhythm rather than pitch adaptations. Stahl and colleagues (53) were able to show that in average, people with aphasia do not benefit from singing over and above rhythmic speech. Moreover, they found that rhythmicity was mostly effective for patients with lesions including the basal ganglia, which is often the case in Broca’s aphasia (63). Interestingly, the rhythmic advantage for speech seems to arise even when hand tapping is absent (53). Furthermore, this element has been reported to be disturbing for the immediate facilitation effect of speech in some cases and not in others (22, 26, 61). Stahl and colleagues (53) found that patients with larger basal ganglia lesions produced more syllables correctly when they were singing or speaking to auditory rhythmic cues (via a metronome). Thus, patients with non-fluent aphasia may critically rely on rhythmic cues but it does not necessarily need to be given in the tactile modality.

It has also been proposed that the effect of the intoned-speech facilitation technique could depend on the rhythmic properties of the spoken language (53, 54). Natural speech in stressed-timed languages (e.g., English, German) has a clearly defined metrical stress pattern, whereas syllable-timed languages (e.g., French) do not have this level of precision. Syllable-timed languages are made of syllables approximately of equal duration. If the rhythmic component is a critical factor that accounts for the facilitation effect of the singing facilitation technique, the rhythmic nature of a subject’s language could also have a role in this effect. On one hand, aphasics speaking a syllable-timed language could benefit from the adjunction of a rhythmical element in speech because rhythmic salience may help the speech segmentation in syllables and words. In TMR (French), the intoned phrases are produced on a melodic rhythm with natural and artificial stresses. The latter are associated with some syllables/words (i.e., function words) that are usually unstressed in normal speech and often omitted in aphasic speech. These additional stresses are thought to help aphasic patients to better produce these words in the intoned sentences. On the other hand, patients whose mother tongue is a stressed-timed language could benefit from the adjunction of a rhythmical element in speech because it may help them to emphasize the natural rhythmic pattern of their language. They could regain more natural prosody that is often disrupted by apraxic symptoms in the speech of non-fluent aphasics. These differential effects remain to be investigated.

Finally, another important aspect of singing is that it slows down articulatory tempo compared to natural speech (54). In fact, singing and rhythmic pacing were found to be similarly effective in slowing down articulatory tempo in patients with motor speech deficits (64) and several studies suggest that longer syllable durations can have a positive effect on speech production (53, 54, 62, 65). Thus, the facilitation effect of the intoned-speech technique could also arise from a syllable duration effect – irrespective of whether singing or rhythmic pacing is used to reduce the articulatory tempo.

### Contribution of musical components in language recovery after MIT (longitudinal studies)

In an efficacy study, Schlaug and colleagues (17) (two subjects) compared the original MIT with a control therapy designed to resemble MIT but without the pitch and rhythmic components. Because MIT led to greater improvement than the control treatment on standard language tests, these components were deemed key efficacy factors for MIT.

Only two longitudinal studies considered differential effects of pitch and rhythm (24, 45). Both used a palliative version of MIT (i.e., an intensive training of a limited set of sentences). Wilson and colleagues (24) used a rigorous single-case design with KL, an experienced musician with chronic severe Broca’s aphasia. KL was trained with a first list of 10 sentences using classical melodic intonation and tapping, and a second list using the rhythmic component but no pitch variation. A third list (control list) was not trained. One week after intervention, KL showed significant improvement in recall and production of the trained sentences compared to the control list, with no significant difference between the two training conditions. It is only 5 weeks after the end of therapy that a more durable effect of combined pitch variation and rhythm was found. According to the authors, this learning condition may promote more efficient memory storage or access to the trained phrases. However, their patient was an experienced musician, such that it is unknown whether the findings generalize to a larger clinical population.

In a group study made of musically naive subjects, Stahl and colleagues (45) compared the production of 15 common sentences trained with a well-known melody in five participants, with rhythm only in five other subjects, or non-trained in a third group of five subjects who underwent standard speech therapy (control group). They found greater improvement in both groups who trained the list of sentences as compared to the control group. No significant difference appeared at the group level between the training conditions after the treatment or at 3-month follow-up. The results provide support for the idea that rhythm may well be the critical component in palliative versions of MIT. Pitch variation did not add any clinical effect over rhythm in this group of patients in contrast to Wilson and colleagues’ case study (24). Wilson and colleagues’ case was an experienced singer whereas none of the participants of Stahl and colleagues had musical training. Thus, pitch variation might only help maintaining therapeutic gains in trained sentences when participants have a significant musical background. This consideration deserves more attention in future studies. In both studies, no transfer to the untrained phrases was significant but patients made considerable progress in the production of trained phrases in a short time, providing support for the hypothesis that palliative versions of MIT can be a suitable choice for some aphasic patients.

To summarize, the rhythmic component appears to be crucial for the immediate facilitation effect of intoned speech, and it also plays a role in the program effect in palliative versions of MIT. The pitch component may maintain therapeutic gains on trained material in palliative versions of MIT but this effect could depend on the patient’s musical background. This echoes what has been demonstrated in the rehabilitation of motor speech problems: some training conditions may drive performance during training, but not during long-term maintenance of learned skills, and vice versa (66). The relative therapeutic effects of rhythm and pitch variation remain to be tested in language recovery using the original MIT. To date, longitudinal studies using original MIT in comparison to a control therapy have not addressed this issue (17, 18, 29, 30). In these studies, the control treatments differ from MIT with regard to both rhythmic and pitch elements. Future studies on the original MIT could set up control treatments in a way that it allows conclusions regarding the relative therapeutic effects of singing, rhythmic speech as well as other therapeutic elements of MIT.

## MIT for Apraxia of Speech

A question rarely raised in the literature is why MIT appears to have a beneficial effect on verbal production in Broca’s aphasia but virtually none on other aphasic syndromes (6). This issue could be directly addressed by examining which symptoms are unique to this aphasia type. Verbal expression in Broca’s aphasia is characterized by anomia (i.e., word-retrieval difficulty), agrammatism (i.e., grammar and syntax deficit), and apraxia of speech (AOS: a motor speech disorder affecting the planning or programing of speech movements) (6, 67). Anomia is the core symptom of aphasia, and is therefore present in all aphasic syndromes. However, agrammatism and AOS are clinical markers used to differentiate Broca’s from other aphasias. MIT has shown little effect on agrammatism (4). Therefore, we propose that MIT might act on AOS.

As defined above, AOS is a deficit in motor planning or programing of speech movements (66, 68, 69). Apraxic patients typically have an automatic-voluntary motor dissociation. Patients with AOS exhibit this dissociation in speech output (similar to the dissociation between non-propositional and propositional language production in Broca’s aphasia). AOS is considered a disorder of speech, not language: the deficit disrupts the translation of a phonological representation into a phonetic representation to be executed by the articulators (68–72). Patients with pure AOS do not have the anomia that characterizes aphasia. They have normal phonological representations and they can express themselves well in writing because they have intact non-speech-language means (73). In contrast, they struggle to produce words: their speech is slow, effortful, and often hard to understand. If MIT acts mainly on the AOS component in Broca’s aphasia syndrome, it could be used as a treatment for motor speech problems, and not for language in the strict sense.

Even though efficacy studies on MIT have shown progress in standard test scores on language abilities, it is nevertheless possible that language competence does not improve so much, but instead that reductions in the AOS component of Broca’s aphasia allow this competence to be better expressed. For example, patients with Broca’s aphasia might fail a picture-naming test due to word-retrieval impairment (anomia) or because they cannot produce the correct word even though they know it (AOS). AOS can be masked by aphasia (73). Although clinicians use their clinical judgment to differentiate the two conditions, AOS has not received as much attention as language deficit in aphasia, and reliable assessment tools to disentangle the two deficits are lacking (66). Thus, improved scores on standard language tests following MIT might be due to motor speech improvement.

A number of treatments for AOS [see the treatment guidelines for AOS published by Wambaugh et al. (74)] recommend techniques that resemble those used in melodic-speech therapies. They include singing (49), hand tapping paired with word or sentence production (75, 76), control of speech rate by encouraging prolonged speech production, either alone (77) or in synchrony with a metronome (75, 78), or with rhythmic beep sequences matching the normal rhythm of each sentence [Metrical Pacing Technique; (79)]. Although singing, hand tapping, synchronization, or rhythmic and speech rate control are included in MIT, the program has not been tested as a treatment for AOS. We believe that this issue merits investigation. With the advance of available techniques to investigate motor speech deficits (66), future studies could explore this issue more deeply in melodic-speech therapies for patients with Broca’s aphasia.

## Conclusion

The renewed interest in MIT-like interventions should provide new opportunities to clarify aspects that have important clinical implications for patients. Therapeutic protocols using singing as a speech facilitation technique are not necessarily MIT. Among the protocols that have been considered as MIT, we distinguish in fact three main types of treatment based on different therapeutic goals: original MIT, TMR, and palliative MIT. The original MIT aims to restore propositional speech through a functional reorganization of language production. MIT variations such as TMR train patients to use a facilitation technique in case of speech struggle, while palliative versions of MIT help patients with the most severe expressive deficits produce a limited set of useful, readymade phrases.

The treatment mechanisms may depend on the therapeutic approach. Future studies could more clearly distinguish between the facilitation effect of a technique and the therapeutic effect of a treatment. Currently, the mechanisms of the intoned-speech technique appear to involve the rhythmic component of the melody along with left hemisphere peri-lesional regions. However, the brain correlates of MIT’s therapeutic effect on language recovery are still unclear, even at the gross level of brain hemispheric lateralization. The combination of rhythm and pitch variation seems to be key elements in the original program but the relative role of rhythm and pitch in MIT’s therapeutic effect has not been tested yet. Moreover, other components of the treatment could also have a significant impact on language and/or speech recovery, especially in TMR and palliative variations of MIT. This deserves investigation in order to improve the efficacy of speech and language therapy in different clinical profiles of patients with non-fluent aphasia.

Although MIT is regarded as a language treatment for Broca’s aphasia, it could also act on motor speech deficits in this aphasic syndrome. Future experimental studies could explore this issue more thoroughly.

## Conflict of Interest Statement

The authors declare that the research was conducted in the absence of any commercial or financial relationships that could be construed as a potential conflict of interest.
